# Effects of Functionalized Materials and Bacterial Metabolites on Quality Indicators in Composts

**DOI:** 10.3390/ma15238564

**Published:** 2022-12-01

**Authors:** Krzysztof Gondek, Piotr Micek, Monika Mierzwa-Hersztek, Jerzy Kowal, Krzysztof Andres, Katarzyna Szczurowska, Marcin Lis, Krzysztof Smoroń

**Affiliations:** 1 Department of Agricultural and Environmental Chemistry, University of Agriculture in Krakow, Al. Mickiewicza 21, 31-120 Krakow, Poland; 2 Department of Nutrition, Animal Biotechnology and Fisheries, University of Agriculture in Krakow, Al. Mickiewicza 24/28, 30-059 Krakow, Poland; 3 Department of Zoology and Animal Welfare, University of Agriculture in Krakow, Al. Mickiewicza 24/28, 30-059 Krakow, Poland; 4 Department of Animal Reproduction, Anatomy and Genomics, University of Agriculture in Krakow, Al. Mickiewicza 24/28, 30-059 Krakow, Poland; 5 Specialized Mining Company “Górtech” sp. z o. o., ul. Wielicka 50, 30-552 Krakow, Poland

**Keywords:** compost, poultry litter, functionalized materials, bacterial metabolites

## Abstract

The addition of functionalized materials (biochar, zeolite, and diatomite) and lyophilized metabolic products of *Pseudomonas* sp. and *Bacillus subtilis* to composted biomass may bring many technological and environmental benefits. In this study, we verify the effects of biochar, zeolite Na-P1 (Na_6_Si_10_Al_6_O_32_·12 H_2_O), diatomite (SiO_2__nH_2_O), and bacterial metabolites on the composting of biomass prepared from poultry litter, corn straw, grass, leonardite, and brown coal. The experimental design included the following treatments: C—biomass without the addition of functionalized materials and bacterial metabolites, CB—biomass with the addition of biochar, CBM—biomass with the addition of biochar and bacterial metabolites, CZ—biomass with the addition of zeolite, CZM—biomass with the addition of zeolite and bacterial metabolites, CD—biomass with the addition of diatomite, and CDM—biomass with the addition of diatomite and bacterial metabolites. Composts were analyzed for enzymatic and respiratory activities, mobility of heavy metals, and the presence of parasites. The results of this study revealed that, among the analyzed functionalized materials, the addition of diatomite to the composted biomass (CD and CDM) resulted in the most effective immobilization of Cd, Zn, Pb, and Cu. Zinc immobilization factors (IFHM) for diatomite-amended composts averaged 30%. For copper, each functionalized material was found to enhance mobilization of the element in bioavailable forms; similar observations were made for lead, except for the compost to which biochar and bacterial metabolites were added (CBM). The determined values of biochemical indicators proved the different effects of the applied functionalized materials and bacterial metabolites on the microbial communities colonizing individual composts. The dehydrogenase activity (DhA) was lower in all combinations as compared with the control, indicating an intensification of the rate of processes in the studied composts. The highest basal respiration (BR) and substrate-induced respiration (SIR) activities were determined in composts with the addition of bacterial metabolites (CBM, CZM, and CDM). The addition of functionalized materials completely inactivated *Eimeria* sp. in all combinations. In the case of *Capillaria* sp., complete inactivation was recorded for the combination with zeolite as well as biochar and diatomite without bacterial metabolites (CB, CZ, and CD).

## 1. Introduction

Composting is an aerobic process in which organic materials are degraded and transformed by microorganisms into organic and inorganic products [1,2]. Composting is a way to transform organic materials, including waste from crop and livestock production, into products that can be safely and profitably used in the environment for soil fertilization, reclamation of degraded land, or revitalization of brownfields [2,3]. Composts can also play a fungistatic role in the soil environment for the benefit of crop health [4].

The quantitative and qualitative composition of microbial populations during composting characterizes their ability to degrade composted biomass [5]. Changes in the quantitative and qualitative composition of microbial populations during composting depend on the qualitative composition of biomass, the availability of nutrients, and the environmental conditions. The dominant groups of microorganisms in the composting process are bacteria and fungi. The type of organic materials used and the quantitative and qualitative composition of the microbial population involved in composting strongly influence compost quality [6,7]. Compost quality considered from the chemical, biochemical, and sanitation point of view is a key element that indicates process efficiency, and also determines possible directions of compost management. The amount of waste in the world is enormous, and it should be noted that nearly 50% of this waste contains organic matter which mainly includes food waste, waste from animal and agricultural production, waste from the agro-food industry, and wood waste [8]. Given such a rich resource of organic materials to use, designing the composition of biomass for composting should not be problematic. However, it should be stressed that the size and type of organic material resources are not the only elements that determine a successful composting process. According to Sokač et al. [9], the operational efficiency of the composting process depends on numerous variables such as temperature, pH, moisture content, and the C/N ratio. The cited authors have stated that in order to achieve optimal composting efficiency, all variables and their relationships must be considered at the same time.

Currently, many researchers are working to develop integral composting models capable of describing and predicting all physicochemical and biochemical changes that occur during the biological transformation of waste [9]. At the same time, there is research being conducted to identify alternative additives to use in composted biomass to optimize the biochemical processes taking place during waste conversion. The research on alternative additives has mainly focused on materials that would improve the environmental conditions for microbial growth. These materials should be characterized by: the ability to optimize process efficiency; favorable influences on the physical properties of composted biomass (porosity, specific surface area, and cation exchange capacity); the ability to effectively reduce ammonia (NH_3_), nitrous oxide (N_2_O), and methane (CH_4_) emissions; the bioavailability of toxic trace elements; as well as health risks.

The use of various types of additives, even in small amounts, can significantly modify the composting process [10]. As shown by studies to date, mineral, organic, and biological additives stimulate microbial activity, thus, altering the duration of individual composting phases. According to Himanen and Hänninen [11], the duration of the thermophilic phase increased from 2 to 3 weeks after adding commercial products containing zeolite, kaolinite, chalk, ash, and sulfates to composted biomass. Many studies have reported rapid temperature increases in composted animal manure, food waste, or plant waste following the addition of mineral and organic materials [12,13,14]. The faster temperature increase when biomass is enriched with various materials may result from an increase in the microbial population, and thus, a higher intensity of the biochemical processes taking place. As stated by Waqas et al. [14], as compared with the control, composted materials supplemented with biochar reached the thermophilic temperature faster and increased organic matter degradation by 14.4–15.3%, the concentration of NH_4_^+^ by 37.8–45.6%, and the concentration of NO_3_^−^ by 50–62%. However, on the one hand, it should not be assumed that the addition of each material will cause changes in the temperature profile of the composting process [15,16]. On the other hand, the parameters that may change are the chemical composition and the value of sanitation indicators in composts [6,17,18].

Supplementation of composted biomass with functionalized materials (before setting up the composting process) should be an indispensable element of the design, and also the management of composting in terms of the environmental impact of the process and product in terms of climate neutrality. Therefore, it is important to search for functionalized materials with a broader spectrum of action, going beyond the process itself, i.e., acting after the compost is introduced into the soil. The effects of adding biochar, zeolite, and diatomite on composting, as well as some chemical and biological indicators of the product have already been described in the literature [18,19,20]. However, there is still no information on the synergistic effect of functionalized materials such as biochar, zeolite Na-P1 (Na_6_Si_10_Al_6_O_32_·12 H_2_O), diatomite (SiO_2__nH_2_O), and biologically active substances, i.e., surfactants and siderophores, which are products of microbial metabolism. Designing composted biomass to contain materials of very different origins and functions may bring many measurable technological (e.g., facilitating the proper structure of composted biomass, maintaining adequate humidity and better air exchange, and appropriate pH) and environmental (e.g., using a wide range of waste materials with different organic matter decomposition rates, better conditions for carbon sequestration, immobilization of heavy metals, and hygienization of treated waste) benefits. Additionally, the addition of bacterial metabolites such as siderophores and surfactants to the composted biomass is of great significance.

Surfactants or surface active agents are a group of substances with specific properties. Such compounds consist of two parts: one portion is hydrophilic and the other portion is hydrophobic. The hydrophilic, polar portion shows high affinity to water and polar liquids, and also has lyophobic properties. Siderophores are organic compounds with low molecular masses that are produced by microorganisms and plants growing under low iron conditions. The primary function of these compounds is to chelate the ferric iron [Fe(III)] from different terrestrial and aquatic habitats, and thereby, make it available for microbial and plant cells. Siderophores have received much attention in recent years because of their potential roles and applications in various areas of environmental research. Their significance in these applications is because siderophores have the ability to bind a variety of metals in addition to iron, and they have a wide range of chemical structures and specific properties. For instance, siderophores function as biocontrols, biosensors, and bioremediation and chelation agents, in addition to their important role in weathering soil minerals and enhancing plant growth [21]. Xi et al. [22] argued that the addition of biosurfactant to solid waste containing Hg and sodium pentachlorophenolate could effectively improve composting by removing both contaminants faster. According to Gong et al. [23], the addition of rhamnolipid biosurfactant and/or microorganisms alone or in all combinations significantly increased the growth rate of *E. fetida* as well as the number of juveniles and cocoons. It also resulted in denser populations of cellulolytic fungi and Azotobacter and increased cellulase and urease activities in vermicomposts [23]. O’Brien et al. [24] and O’Brien and Buckling [25] reported that siderophores could act as public foods by inactivating toxic heavy metals, in which case, they bind to metals, preventing their uptake by cells and rendering the metal (and thus the environment) non-toxic [24,26]. Hesse et al. [27] also reported on the detoxifying role of siderophores. However, the available literature still lacks information on whether the addition of functionalized materials and bacterial metabolites to composted biomass can reduce animal endoparasites or their developmental forms.

Given the above, it was hypothesized that the combined use of functionalized materials (biochar, zeolite, and diatomite) and microbial metabolic products (siderophores and surfactants) has synergistic effects and (i) reduces heavy metal mobility, (ii) optimizes biochemical processes, and (iii) improves the sanitation condition of poultry litter composts.

## 2. Materials and Methods

### 2.1. Materials Used for Composting and Process Conditions

This study was carried out under laboratory conditions (air temperature in the room 20 ± 2 °C) on composts prepared from poultry litter, corn straw, fresh grass, leonardite, and brown coal. The proportions of components are given in Table 1.

The composted biomass was supplemented with biochar, synthetic zeolite Na-P1, and diatomite after calcination. The addition of functionalized materials was 5% (relative to the dry matter of the mixture). Lyophilized metabolic products of *Pseudomonas* sp. and *Bacillus subtilis* (mixed in a 1:1 ratio) were also added to the respective combinations at 0.400 g (Σ lyophilized metabolic products of *Pseudomonas* sp. and *Bacillus subtilis*). The lyophilisate contained the transformation products of microorganisms in the amount of at least 10^8^ cfu/g.

Poultry litter came from a domestic guinea fowl farm. The birds were kept in a floor system on peat bedding at a stocking rate of 6 birds/m^2^. Peat was spread on the floor at 6 L per 1 m^2^ once before introducing the guinea fowl. The animals were fed an industrial compound feed containing 14.9% crude protein, 5.1% crude fat, and 4.6% crude fiber. The compound feed consisted of wheat, corn, wheat bran, soybean oil, and post-extraction soybean and sunflower meal. Corn straw came from the field cultivation of corn for grain, while the fresh grass was from green space maintenance. Brown coal used in the study came from the output of Kopalnia Węgla Brunatnego Sieniawa Sp. z o.o. (Brown Coal Mine Sieniawa, Poland). Biochar was produced by Carbon Team (Poland) from coniferous tree waste at 450 °C. Diatomite after calcination (750–800 °C) was produced by Specjalistyczne Przedsiębiorstwo Górnicze GÓRTECH Sp. z o.o. (Poland). In addition, synthetic zeolite Na-P1 was produced by hydrothermal method from fly ash generated from conventional coal combustion at the Kozienice Power Plant. Bacterial metabolites were obtained from RDLS Sp. z o.o., and their production process is described in the publication and patent application [28,29]. Selected chemical parameters of the materials used for composting are given in Table 2.

Oocysts and parasite eggs were found by the McMaster method in the feces of the litter used for the study. Contamination with developmental forms of parasites in the feces of the litter used for the study was as follows (the sum of oocysts and eggs per gram): *Eimeria* sp., 20; *Heterakis* sp., 40; and *Capillaria* sp., 40.

Composting was carried out for 9 weeks (from mid-July to the first week of September 2021). The process was conducted in a 0.8 × 0.9 × 0.6 m bioreactor; perforated-wall containers holding 6.50 kg of fresh composted material were placed into it. The bioreactor chamber was isolated from external conditions and aerated at 0.015 m^3^ min^−1^, 6 times a day. For better aeration and homogenization, the composted biomass was taken out of the bioreactor once a week and mixed by hand.

The experimental design included the following treatments: C—biomass without the addition of functionalized materials and bacterial metabolites, CB—biomass with the addition of biochar, CBM—biomass with the addition of biochar and bacterial metabolites, CZ—biomass with the addition of zeolite, CZM—biomass with the addition of zeolite and bacterial metabolites, CD—biomass with the addition of diatomite, CDM—biomass with the addition of diatomite and bacterial metabolites.

### 2.2. Chemical Analyses in Materials and Composts

In the materials used for composting and in composts, the pH was determined potentiometrically in a suspension of compost and water (compost/water = 1:5) with a pH-meter (CP-505), and electrical conductivity (EC) (compost/water = 1:5) with a conductivity meter (conductivity/oxygen meter CCO-501). The C and N contents in composts were determined using a CNS analyzer (Vario MAX Cube, Elementar) [30]. Total contents of the studied elements were determined in composts after digesting the samples in concentrated HCl and HNO_3_ (3:1 *v/v*) in a microwave oven. The total digestion time was 30 min, the maximum digestion temperature was 240 °C, and the maintained pressure was 40 bar. Mobile forms of Cu, Cd, Pb, and Zn were extracted from composts with redistilled water (compost/water = 1:10) for 2 h. The elemental contents in the filtrates and extracts were determined using an argon inductively coupled plasma optical emission spectrometer (ICP-OES Optima 7300DV, Perkin Elmer). Heavy metal immobilization factors (IFHM) in the composted biomass were calculated from the following equation [31]:

(1) $$\mathrm{Immobilized}\;\mathrm{metal}\;\left(\%\right)=\frac{\left(\mathrm{water}\;\mathrm{metal}\;\mathrm{for}\;\mathrm{the}\;\mathrm{control}-\mathrm{water}\;\mathrm{metal}\;\mathrm{for}\;\mathrm{the}\;\mathrm{treated}\;\mathrm{sample}\right)\times 100}{\mathrm{water}\;\mathrm{metal}\;\mathrm{for}\;\mathrm{the}\;\mathrm{control}}$$

### 2.3. Biochemical Analyses in Composts

The basal respiration (BR) was determined using the method according to ISO 16072 [32]. Moist compost subsamples (20 g) were incubated at 20 ± 2 °C for 24 h. The released CO_2_ was absorbed in 0.05 M NaOH solution and precipitated as barium carbonate by adding 0.5 M BaCl_2_ solution. Non-consumed sodium hydroxide was titrated with 0.1 M HCl in the presence of phenolphthalein as an indicator and the amount of CO_2_ was calculated [32]. The substrate-induced respiration (SIR) data were determined by CO_2_ evolution 6 h after adding glucose (10 g kg^−1^ aqueous solution) [33]. The respiratory-activation quotient (QR) was calculated by dividing the BR rate by the SIR rate according to ISO 17155 [34].

The dehydrogenases activity (DhA) activity was determined by converting colorless, water-soluble 2,3,5-triphenyltetrazolium chloride (TTC) into red-water-insoluble 1,3,5-triphenylformazan (TPF). Samples (5 g) were incubated at 37 ± 2 °C for 24 h. The enzymatic activity of dehydrogenases was determined by colorimetric analysis using a Backman DU 640 spectrophotometer at a wavelength of 485 nm.

### 2.4. Parasitological Analyses in Composts

Parasitological analyses were performed in composts with natural water content. The presence of *Eimeria* sp. oocysts and *Capillaria* sp. eggs was tested in 100 g samples using the method developed by Dada (1979). The determination involved mixing 100 g of compost with 200 mL of 5% NaOH solution. Then, the samples were allowed to stand for 1 h, shaken for 20 min, and transferred to 50 mL centrifuge tubes, straining the suspension through a 1 mm mesh sieve. After centrifugation (3 min, 1500 rpm), the solution from above the precipitate was decanted, and water was poured over the precipitate. The samples were centrifuged again, and the remaining precipitate was mixed and flooded with flotation fluid (saturated solution of NaCl and sucrose, d = 1.26 g mL^−1^). After re-centrifugation and refilling the flotation fluid to the convex meniscus, a cover slip was placed over the test tube. After 20 min, the cover slips were transferred from the test tubes to glass slides with a drop of glycerol. The developmental forms of parasites were searched for in the resulting preparations under the microscope at 100× and 400× magnifications.

### 2.5. Statistics

The experiment was carried out in a completely randomized design with three replicates. One-way analysis of variance (ANOVA) was carried out using Statistica 12.0 (TIBCO Software Inc., Palo Alto, CA, USA). The significance of differences between the means was conducted using a Duncan test at *p* ≤ 0.05. Variability of results within the treatments was discerned by calculating standard deviation (SD).

## 3. Results

### 3.1. Temperature Changes during Composting

**Figure 1:**
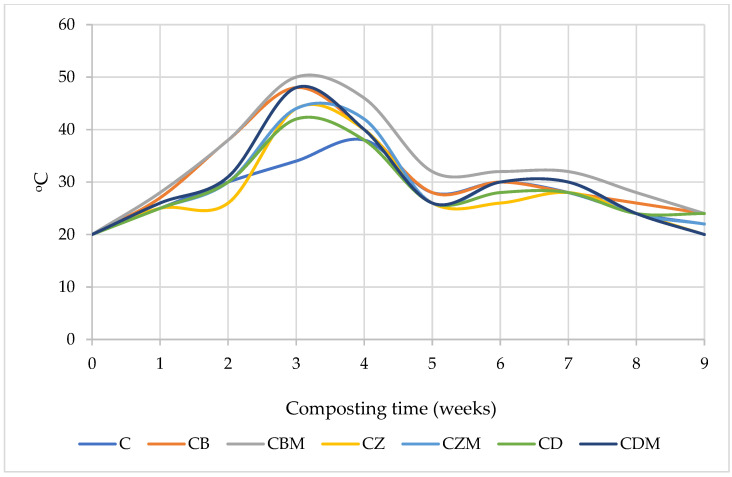
Temperature changes during composting. C—biomass without the addition of functionalized materials and bacterial metabolites, CB—biomass with the addition of biochar, CBM—biomass with the addition of biochar and bacterial metabolites, CZ—biomass with the addition of zeolite, CZM—biomass with the addition of zeolite and bacterial metabolites, CD—biomass with the addition of diatomite, CDM—biomass with the addition of diatomite and bacterial metabolites.

The temperature of composted biomass increased most rapidly in treatments with the addition of biochar (CB) and biochar and bacterial metabolites (CBM) (Figure 1). The highest temperatures were recorded in the third week of the process in the same treatments and in the treatment with the addition of diatomite and bacterial metabolites (CDM). The lowest temperature was determined for the control biomass (C), where the maximum temperature was reached with a week delay (beginning of the 4th week of composting). Regardless of the treatment, the temperature range of the composted biomass was generally typical for this type of process. However, it should be noted that during the whole composting period, the highest temperature, as compared with other treatments, was recorded for CBM.

### 3.2. Effects of Functionalized Materials and Bacterial Metabolites on Selected Chemical Properties of Composts

The content of dry matter in composts did not differ significantly (Table 2). The greatest amount of dry matter was determined in composts with the additions of diatomite (CD) and diatomite and bacterial metabolites (CDM).

Composts supplemented with biochar (BC) and biochar and bacterial metabolites (CBM) contained the significantly highest amount of carbon (Table 3). Lower carbon contents were determined in other composts as compared with the control (C). The reduction in carbon content averaged 11.1% in composts with zeolite (CZ, CZM) and over 20.5% in composts with diatomite (CD, CDM) as compared with the control compost. In general, the nitrogen content was significantly lower in all composts as compared with C (Table 3). The lowest N loss was observed in CB and CBM composts (average 3.3%).

The significantly lowest pH value was determined in compost C (Table 3). The addition of biochar and diatomite to the composted biomass had a comparable effect on the pH of composts, shaping it at a value of about 7.60. The pH values recorded for CZ and CZM composts were over 0.2 higher.

The electrical conductivity (EC) value in the C compost was 4.36 mS cm^−1^ (Table 3). The significantly highest EC value was determined in the CBM compost (4.60 mS cm^−1^). The addition of both zeolite and diatomite rendered the EC values of composts significantly lower as compared with biochar-enriched composts, regardless of the supplementation of composted biomass with bacterial metabolites.

### 3.3. Effects of Functionalized Materials and Bacterial Metabolites on the Heavy Metal Content in Composts

**Figure 2:**
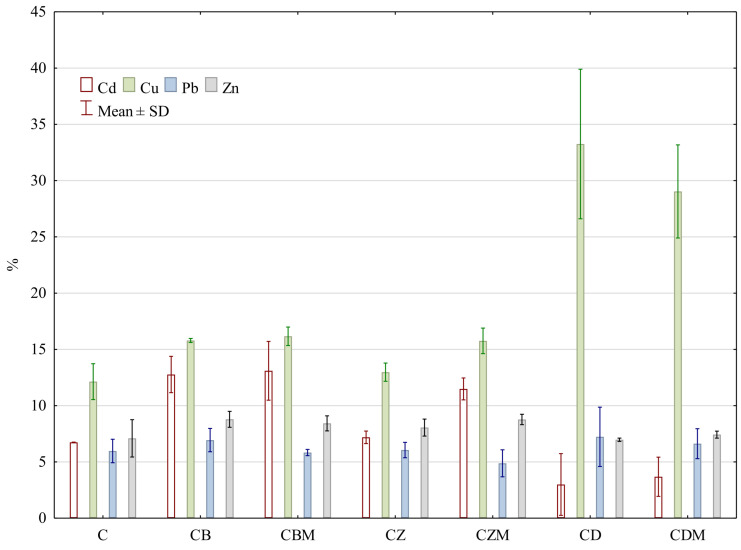
Share of water-extracted heavy metals in the total heavy metal content in composts. C—biomass without the addition of functionalized materials and bacterial metabolites, CB—biomass with the addition of biochar, CBM—biomass with the addition of biochar and bacterial metabolites, CZ—biomass with the addition of zeolite, CZM—biomass with the addition of zeolite and bacterial metabolites, CD—biomass with the addition of diatomite, CDM—biomass with the addition of diatomite and bacterial metabolites.

**Figure 3:**
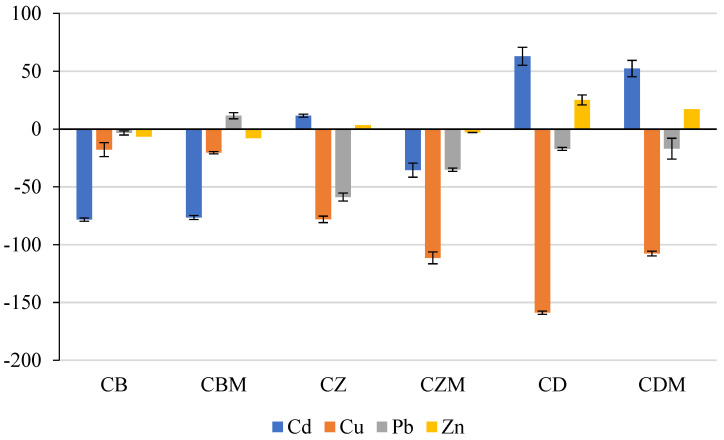
Value of the heavy metal immobilization factor (IFHM) in composts. C—biomass without the addition of functionalized materials and bacterial metabolites, CB—biomass with the addition of biochar, CBM—biomass with the addition of biochar and bacterial metabolites, CZ—biomass with the addition of zeolite, CZM—biomass with the addition of zeolite and bacterial metabolites, CD—biomass with the addition of diatomite, CDM—biomass with the addition of diatomite and bacterial metabolites.

The content of bioavailable forms of heavy metals differed not only by the type of the analyzed element, but also by the functionalized additive introduced into the composted biomass (Table 3). Significantly, most numerous bioavailable forms of cadmium were recorded in CB and CBM composts. The determined contents were also reflected in the highest share of these cadmium forms in the total element content (Figure 2). As compared with other composts, the contents of bioavailable cadmium in CD and CDM were significantly the lowest. However, it should be noted that a better effect in reducing cadmium bioavailability was observed in the combination without bacterial metabolites. The positive effect of diatomite addition on cadmium immobilization was also confirmed by the immobilization factor (IFHM) values determined for CD and CDM composts (Figure 3).

The content of bioavailable copper was significantly higher in composts supplemented with zeolite and diatomite as compared with C, CB, and CBM composts (Table 3). A significant share of bioavailable copper in its total content was observed for CD and CDM combinations, totaling more than 33% and 29%, respectively (Figure 2). It is noteworthy that in the treatments enriched with biochar and zeolite, the share of bioavailable copper in their total contents increased in CBM and CZM as compared with CB and CZ. Taking the control treatment (C) as a reference, each functionalized material was found to deteriorate the copper immobilization conditions (Figure 3).

The content of bioavailable forms of lead was lowest in C, CB, and CBM composts (Table 3). The content of bioavailable lead increased the most in the CZ and CZM composts as compared with the control. There were no significant differences in the share of bioavailable lead in its total content for all functionalized materials added (Figure 2). The immobilization factor IFHM values confirmed that the addition of zeolite and diatomite to the composted biomass increased the potentially bioavailable forms of this element (Figure 3).

The contents of bioavailable forms of zinc were similar, except for CD and CDM composts (Table 3). The addition of diatomite to the composted biomass, especially in the combination without bacterial metabolites (CD), significantly reduced the content of bioavailable zinc. The share of bioavailable forms of lead in the total content did not show much variation and ranged from 6.97% to 8.79% (Figure 2). The lowest contents of bioavailable zinc were reflected in IFHM values, which indicated a clear element immobilization for CD and CDM composts (Figure 3).

### 3.4. Effects of Functionalized Materials and Bacterial Metabolites on Selected Biochemical Properties of Composts

Dehydrogenase activity (DhA) is related to the transformation of carbon and nitrogen compounds. The highest DhA was observed in the control compost (Table 4). Supplementation of the composted biomass decreased DhA, most in CBM and CD composts. Of all the functionalized materials, zeolite, when added to composted materials, had the best effect on DhA, with both CZ and CZM showing the least reduction in DhA as compared with C. As compared with the control, the decrease in DhA activity was 11.9% in CZ and 2.5% in CZM.

The basal respiration (BR) value determined in the study differed significantly among the composts. The highest BR value, as compared with the C, was found in the compost with the addition of zeolite and bacterial metabolites (CZM) (Table 4). Other composts showed lower BR values.

The addition of glucose to the analyzed compost samples significantly increased the basal respiration rate, which was caused by the appearance of a source of readily available carbon in the living environment of microorganisms. It was found that the greatest effect on the SIR value had the addition of biochar, especially biochar and bacterial metabolites (CBM), to the composted biomass. The greatest increase in the SIR value was determined in the CBM compost (as compared with the C compost, the value increased by over 22%).

The calculated respiratory-activation quotient (QR), reflecting the amount of microorganisms present in the dormant or active state, ranged from 0.347 for CBM to 0.525 for CZ (Table 4).

### 3.5. Effects of Functionalized Materials and Bacterial Metabolites on Developmental Forms of Selected Parasites

*Eimeria* sp. oocysts and *Capillaria* sp. eggs were found in the tested compost samples, but no *Heterakis* sp. eggs, which were earlier present in the feces of birds, were found. *Eimeria* sp. coccidia and the highest number of *Capillaria* sp. eggs were found in the C compost (Table 5). CBM and CDM were characterized by the lowest number of *Capillaria* sp. eggs. No *Capillaria* sp. eggs were found in the CZM compost.

## 4. Discussion

The problem of proper waste management has recently become a global issue. It affects practically every environmental sphere and poses a threat to every form of life on earth. The numerous processes and technologies used for waste disposal and transformation are powerful tools to manage waste. However, changing trends and production and environmental conditions are forcing the search for new or the improvement of currently used waste processing technologies. Composting represents the group of processes termed as environmentally friendly. Currently, many researchers are working to develop integral composting models capable of describing and predicting various physicochemical and biochemical changes that occur during the biological transformation of waste [9]. However, this is not the only field where intensive research work is being carried out. At the same time, there is a search for alternative additives to composted biomass to optimize biochemical processes taking place during waste conversion. In this study, functionalized organic and mineral additives were introduced into a multi-material composition with the main share of poultry litter. Biochar, synthetic zeolite, and diatomite after calcination were used for composting. Metabolites of *Pseudomonas* sp. and *Bacillus subtilis* served as biologically active substances. The introduced materials affected the composting process in different ways, which was also demonstrated by Barthod et al. [10]. Considering the temperature criterion, the temperature reached and the rate of temperature change during the initial composting phase both indicate the greatest reduction of organic matter in biomass supplemented with biochar, especially with the addition of biochar and bacterial metabolites [35]. Other combinations showed a milder temperature profile as well as a lower maximum temperature of the composted biomass. As stated by Waqas et al. [14], composted materials enriched with biochar, irrespective of its production temperature, reached the thermophilic temperature faster than the control. The opposite thesis was presented by Malinowski et al. [36], who found that the addition of biochar to the organic fraction of municipal solid waste did not alter the composting process. The study by Guo et al. [37] on the composting of pig manure with rice straw showed that materials enriched with biochar tended to loosen during composting, and biochar increased the total porosity of compost aggregates by about 90% as compared with other treatments, which was mainly attributed to decomposition effects. Therefore, it can be concluded that the addition of biochar to composted biomass refines its physical structure, and the synergistic effect of biochar and bacterial metabolites can significantly improve the living conditions of microorganisms involved in the process, which is reflected in the temperature—a measurable effect of microorganisms’ activity. The introduction of mineral functionalized materials (zeolite and diatomite) to the composted biomass, even enriched with bacterial metabolites, caused the maximum biomass temperature to take lower values during composting.

The pH values of the obtained composts ranged from 7.54 to 7.87 and were higher for the combination with synthetic zeolite, which had the highest pH value among the functionalized materials used in this study. A relatively high pH in all combinations used in this study may be due to the mineralization of nitrogen compounds, including those derived from litter, which mainly concerns the release of ammonia and ammonium ion [38].

Electrical conductivity (EC) is an important element that determines the natural use of compost. This property can have a significant impact on seed germination as well as plant growth and development. As a result of the high concentration of easily soluble salts, for example, in the soil solution, water uptake by the plant root system may be reduced after applying compost. The effect of microbial decomposition of organic materials is the release of, inter alia, calcium or magnesium ions which increase the electrical conductivity of the compost [38]. In the present study, the addition of both diatomite and zeolite, regardless of the enrichment of biomass with bacterial metabolites, significantly reduced the EC value of composts as compared with the control and biochar combinations, which resulted from the unique meso-microporous structure of both materials and, consequently, their high sorption capacity. Salinity reduction in zeolite-enriched composts was reported by Soudejani et al. [39].

The obtained C/N ratio values indicated that the composted waste was effectively degraded. These values, especially for the combination with functionalized mineral materials (zeolite and diatomite) may suggest that the composting process was carried out too long. It should be noted that the addition of bacterial metabolites was of little importance here. Compost amended with biochar proved to be more stable in terms of the C/N ratio. Biochar contains carbon structures that are difficult to decompose. According to Barthod et al. [10], biochar, as a highly aromatic pyrolysis product, is stable when added to composting, and therefore, can increase the carbon sequestration potential, contributing to climate change mitigation.

Heavy metals in compost, and especially their bioavailable forms, can have adverse effects on plant growth and development, and consequently, on animal and human health [38]. Therefore, determining both the content and bioavailability of heavy metals is important for assessing the quality of composts made from waste substances, including those of animal or municipal origin [10]. During composting, the transformation of organic matter (which receives most attention) takes place and also involves changes in the quantity and speciation of heavy metals, which very often lead to excessive mobilization of unavailable forms of heavy metals. The authors of the present study hypothesized that the use of functionalized materials as well as siderophores and surfactants in composting would reduce the content of water-soluble forms of cadmium, copper, lead, and zinc, a thesis that has not been fully confirmed. The addition of diatomite had a positive effect on the immobilization of cadmium and zinc; for copper, each functionalized material was found to deteriorate the conditions of element immobilization; similar observations were made for lead except for the compost supplemented with biochar and bacterial metabolites. These results proved that there are specific conditions that should be met to limit the bioavailability of heavy metals, where the chemical behavior of the element in such a complex matrix as compost is not without significance. On the one hand, when using biochar as an additive, differences in the binding of heavy metals may relate, for example, to the conditions in which the material was produced, which in turn determines the number of surface functional groups capable of binding heavy metals. On the other hand, when using mineral additives in composted biomass, the effect of heavy metal immobilization is mainly based on adsorption and ion exchange processes.

Modification of composting by introducing various organic or mineral additives affects the microbial communities inhabiting the composted biomass through the influence of these materials on the pH, moisture content, or aeration of the pile, as well as nutrient content [10]. The obtained value of biochemical indicators in the obtained composts proved different effects of the applied functionalized materials on microbial communities. The determined dehydrogenase activity (DhA) value in all combinations was lower than that determined in the control compost; in the case of basal respiration (BR), a slightly higher parameter value was recorded for the compost amended with zeolite and bacterial metabolites; for substrate-induced respiration (SIR), a higher value was determined in the compost with the addition of biochar and bacterial metabolites. The obtained results suggest that, despite many studies on the subject, the high variability in the composition of biomass used for composting, as well as the specific properties of functionalized materials make it very difficult to clearly predict the phases and effects of the process. Functionalized materials such as biochar, due to their properties (mainly porous structure), can increase microbial activity by affecting the moisture content and aeration of the composted biomass [10,14], which in turn impacts the temperature profile. Mineral functionalized additives such as zeolites or diatomite can fulfill their functions in terms of, for example, binding or neutralization of contaminants, while their beneficial effect on microbial activity in nutrient supply may be limited [40,41].

Barthod et al. [10] stated that mineral additives could indirectly alter microbial activity, while the addition of microbial consortium directly changed a microbial community and compost activity. As reported by Sasaki et al. [42], a commercial microbial additive changed the temperature profile of composting and ammonia emissions due to the growth of mesophilic and thermophilic bacteria as compared with normal composting without additives. In this study, no live cultures of microorganisms were used, but their metabolic products (surfactants and siderophores) instead, which did not significantly affect the biochemical parameters of composts.

Metabolic reactions during aerobic transformation of waste lead, among other things, to an increase in the temperature of the composted material. That has a significant effect on the type, number, and species of microorganisms involved in the biological process, but also inactivates most pathogens [43]. According to Hassen et al. [44] and Polprasert [45], a temperature of 55–60 °C maintained for at least 24 h at about 50% humidity in a compost pile is crucial for the removal of most pathogens. In our study, inactivation of *Eimeria* sp. was confirmed in all combinations as compared with the control compost. In the case of *Capillaria* sp., a decrease in the number of eggs was found in the composts with the addition of biochar and bacterial metabolites as well as diatomite and bacterial metabolites, and their complete devastation in the combinations with zeolite and biochar as well as diatomite without bacterial metabolites. Oocysts of *Eimeria* sp. and eggs of *Hetarakis* sp. and *Capillaria* sp. are listed as very and moderately resistant to external factors [46], so their partial or complete elimination in composting with the use of the mentioned additives may indicate efficient hygienization, which is very beneficial from a parasitological point of view. In addition, according to Malinowski et al. [36], the number of potentially pathogenic microorganisms decreased significantly when composting the organic fraction of municipal solid waste with the addition of biochar. Sobrinho et al. [47] determined that most of the composted waste from rabbits, guinea pigs, mice, and hamsters from animal care facilities eliminated more than 90% of Escherichia coli, Salmonella sp., as well as protozoa oocysts and helminth eggs.

## 5. Conclusions

The use of diatomite in composting significantly affected the immobilization of cadmium and zinc; for copper, each functionalized material was found to enhance mobilization of the element in bioavailable forms; similar observations were made for lead, except for the compost to which biochar and bacterial metabolites were added. The determined value of biochemical indicators proved different effects of the applied functionalized materials and bacterial metabolites on the microbial communities colonizing individual composts. The determined activity of dehydrogenases (DhA) was lower in all combinations as compared with the control, indicating a higher rate of processes taking place during composting. Basal respiration (BR) was slightly higher in the compost supplemented with zeolite and bacterial metabolites, while substrate-induced respiration (SIR) was higher in the compost amended with biochar and bacterial metabolites. An increased SIR may suggest that there were no inhibitory substances in that compost or they were in concentrations that did not inhibit the viability of dormant microorganisms. Our results prove a beneficial hygienizing effect of functionalized materials but without the addition of bacterial metabolites. The obtained results do not clearly indicate a synergistic effect of functionalized materials and bacterial metabolites. Appropriate selection of the type of functionalized material and bacterial metabolites, as well as the amount of their supplementation to the composted biomass, requires further studies on different waste materials.

## Figures and Tables

**Table 1:** The share of individual components in biomass for composting.

Material	Composts						
	C	CB	CBM	CZ	CZM	CD	CDM
	Share in Dry Matter (%)						
Poultry litter	50	50	50	50	50	50	50
Corn straw	23	23	23	23	23	23	23
Fresh grass	20	20	20	20	20	20	20
Brown coal	1	1	1	1	1	1	1
Leonardite	1	1	1	1	1	1	1
Biochar	-	5	5	-	-	-	-
Zeolite	-	-	-	5	5	-	-
Diatomite	-	-	-	-	-	5	5
*Pseudomonas* sp. metabolites	-	-	0.20 *	-	0.20 *	-	0.20 *
*Bacillus subtilis* metabolities	-	-	0.20 *	-	0.20 *	-	0.20 *

* g/6.5 kg of biomass; C—biomass without the addition of functionalized materials and bacterial metabolites; CB—biomass with the addition of biochar; CBM—biomass with the addition of biochar and bacterial metabolites; CZ—biomass with the addition of zeolite; CZM—biomass with the addition of zeolite and bacterial metabolites; CD—biomass with the addition of diatomite; CDM—biomass with the addition of diatomite and bacterial metabolites.

**Table 2:** Selected chemical parameters of the materials used for composting (mean ± SD).

Material	Dry Matter	Ash	N	C	pH	EC *	Cd	Cu	Pb	Zn
	g kg^−1^	g kg^−1^ dm				mS cm^−1^	mg kg^−1^ dm			
Poultry litter	795 ± 72	345 ± 30	34.66 ± 1.77	307 ± 7	6.93 ± 0.00	3.99 ± 0.19	0.485 ± 0.031	13.14 ± 0.75	4.86 ± 0.05	96.85 ± 1.08
Corn straw	925 ± 46	85 ± 1	13.62 ± 0.11	438 ± 22	6.13 ± 0.02	3.02 ± 0.080	0.237 ± 0.047	2.93 ± 0.07	1.96 ± 0.53	59.27 ± 1.26
Fresh grass	313 ± 30	94 ± 2	14.75 ± 0.46	442 ± 4	5.83 ± 0.05	3.98 ± 0.16	0.391 ± 0.001	2.91 ± 0.01	2.04 ± 0.33	25.11 ± 0.14
Brown coal	682 ± 47	82 ± 2	8.53 ± 0.02	647 ± 1	4.58 ± 0.28	0.58 ± 0.04	0.133 ± 0.025	1.05 ± 0.01	1.78 ± 0.07	5.37 ± 0.19
Leonardite	598 ± 36	421 ± 14	4.77 ± 0.08	426 ± 9	5.77 ± 0.25	0.32 ± 0.02	0.264 ± 0.007	4.32 ± 0.22	2.57 ± 0.14	69.86 ± 0.97
Biochar	661 ± 72	94 ± 6	6.48 ± 0.36	420 ± 3	7.47 ± 0.16	0.44 ± 0.09	0.247 ± 0.032	5.48 ± 0.14	1.63 ± 0.01	29.06 ± 0.73
Zeolite	970 ± 29	943 ± 1	0.81 ± 0.09	32 ± 1	11.80 ± 0.07	1.86 ± 0.05	0.346 ± 0.028	21.70 ± 0.86	11.71 ± 0.89	35.13 ± 1.89
Diatomite	999 ± 15	999 ± 0	0.39 ± 0.06	<0.001 ± 0	10.02 ± 0.68	0.61 ± 0.03	0.353 ± 0.022	24.38 ± 0.68	12.38 ± 0.97	37.78 ± 2.07
*Pseudomonas* sp. metabolites	-	197 ± 1	68.09 ± 0.11	453 ± 0	-	-	0.051 ± 0.015	0.76 ± 0.21	1.00 ± 0.48	5.49 ± 1.52
*Bacillus subtilis* metabolites	-	344 ± 2	100.21 ± 0.67	336 ± 3	-	-	0.005 ± 0.000	0.64 ± 0.06	0.61 ± 0.42	20.41 ± 3.03

* EC—electrical conductivity.

**Table 3:** Selected chemical properties of composts (mean ± SD).

Compost	Dry Matter	pH	EC	C	N	Cd *	Cu *	Pb *	Zn *
	g kg^−1^		mS cm^−1^	g kg^−1^ dm		mg kg^−1^ dm			
C	470.0 ** ± 44.7 a	7.54 ± 0.01 a	4.36 ± 0.07 b	245.0 ± 2.6 c	27.1 d ± 0.8 d	0.047 ± 0.002 b	2.50 ± 0.14 a	0.463 ± 0.065 a	11.19 ± 0.3 d
CB	475.6 ± 57.1 a	7.58 ± 0.01 b	4.19 ± 0.14 b	326.5 ± 5.5 d	26.7 ± 0.5 cd	0.084 ± 0.004 d	2.95 ± 0.01 a	0.479 ± 0.018 a	11.92 ± 0.2 e
CBM	468.7 ± 45.7 a	7.61 ± 0.02 b	4.60 ± 0.29 c	321.1 ± 15.4 d	25.7 ± 1.6 c	0.083 ± 0.005 d	3.01 ± 0.08 a	0.410 ± 0.054 a	12.07 ± 0.1 e
CZ	457.3 ± 54.9 a	7.87 ± 0.01 c	3.64 ± 0.03 a	217.8 ± 0.6 b	21.3 ± 0.1 a	0.042 ± 0.003 b	4.46 ± 0.17 b	0.736 ± 0.035 c	10.81 ± 0.4 c
CZM	493.0 ± 49.1 a	7.83 ± 0.04 c	3.77 ± 0.08 a	217.9 ± 3.6 b	23.4 ± 0.1 b	0.064 ± 0.000 c	5.29 ± 0.09 c	0.626 ± 0.041 b	11.52 ± 0.0 d
CD	518.0 ± 59.2 a	7.61 ± 0.02 b	3.61 ± 0.08 a	193.8 ± 1.0 a	21.6 ± 0.3 a	0.017 ± 0.008 a	6.47 ± 0.61 d	0.543 ± 0.082 b	8.37 ± 0.1 a
CDM	506.9 ± 51.9 a	7.60 ± 0.00 b	3.76 ± 0.03 a	195.6 ± 5.7 a	21.8 ± 0.7 a	0.023 ± 0.005 a	5.19 ± 0.78 d	0.542 ± 0.010 b	9.26 ± 0.0 b

C—biomass without the addition of functionalized materials and bacterial metabolites, CB—biomass with the addition of biochar, CBM—biomass with the addition of biochar and bacterial metabolites, CZ—biomass with the addition of zeolite, CZM—biomass with the addition of zeolite and bacterial metabolites, CD—biomass with the addition of diatomite, CDM—biomass with the addition of diatomite and bacterial metabolites, EC—electrolytical conductivity. * Contents of bioavailable forms extracted with H_2_ O. ** Each value represents the mean of three replicates ± SD. The different letters within a column indicate a significant difference at *p* ≤ 0.05 according to Duncan’s multiple range tests

**Table 4:** Biochemical indicators of composts (mean ± SD).

Compost	DhA	BR	SIR	QR
	µg TPF g dm h	µg CO_2_ g dm h		
C	508.4 * ± 15.1 d	220.5 ± 3.0 d	465.4 ± 19.8 c	0.475 ± 0.027 bc
CB	433.9 ± 15.5 ab	177.6 ± 5.0 b	465.2 ± 7.5 c	0.382 ± 0.005 a
CBM	416.6 ± 15.1 a	195.8 ± 10.6 c	568.3 ± 4.8 d	0.347 ± 0.048 a
CZ	448.1 ± 8.5 bc	202.5 ± 2.7 c	385.6 ± 4.0 a	0.525 ± 0.001 d
CZM	495.5 ± 6.9 d	222.7 ± 15.9 d	434.1 ± 9.3 bc	0.513 ± 0.026 cd
CD	410.4 ± 11.6 a	155.2 ± 3.4 a	400.3 ± 9.0 ab	0.388 ± 0.017 a
CDM	461.9 ± 21.9 c	196.5 ± 4.9 c	434.3 ± 2.2 bc	0.452 ± 0.009 b

C—biomass without the addition of functionalized materials and bacterial metabolites, CB—biomass with the addition of biochar, CBM—biomass with the addition of biochar and bacterial metabolites, CZ—biomass with the addition of zeolite, CZM—biomass with the addition of zeolite and bacterial metabolites, CD—biomass with the addition of diatomite, CDM—biomass with the addition of diatomite and bacterial metabolites, DhA—dehydrogenases activity, BR—basal respiration, SIR—induced respiration, QR—respiratory-activation quotient. * Each value represents the mean of three replicates ± SD. The different letters within a column indicate a significant difference at *p* ≤ 0.05, according to Duncan’s multiple range tests.

**Table 5:** Developmental forms of parasites found in the tested composts.

Sample	*Eimeria* sp.	*Capillaria* sp.
C	+	++
CB	−	−
CBM	−	+
CZ	−	−
CZM	−	−
CD	−	−
CDM	−	+

The following were found: ++, numerous developmental forms; +, single developmental forms; −, no developmental forms. C—biomass without the addition of functionalized materials and bacterial metabolites, CB—biomass with the addition of biochar, CBM—biomass with the addition of biochar and bacterial metabolites, CZ—biomass with the addition of zeolite, CZM—biomass with the addition of zeolite and bacterial metabolites, CD—biomass with the addition of diatomite, CDM—biomass with the addition of diatomite and bacterial metabolites.

## Data Availability

Not applicable.
